# Prediction of Cyclic Stress–Strain Property of Steels by Crystal Plasticity Simulations and Machine Learning

**DOI:** 10.3390/ma12223668

**Published:** 2019-11-07

**Authors:** Yuto Miyazawa, Fabien Briffod, Takayuki Shiraiwa, Manabu Enoki

**Affiliations:** Department of Materials Engineering, School of Engineering, The University of Tokyo, Tokyo 113-8656, Japan; miyazawa@rme.mm.t.u-tokyo.ac.jp (Y.M.); briffod@rme.mm.t.u-tokyo.ac.jp (F.B.); enoki@rme.mm.t.u-tokyo.ac.jp (M.E.)

**Keywords:** steels, fatigue, cyclic stress-strain curve, crystal plasticity, artificial neural network

## Abstract

In this study, a method for the prediction of cyclic stress–strain properties of ferrite-pearlite steels was proposed. At first, synthetic microstructures were generated based on an anisotropic tessellation from the results of electron backscatter diffraction (EBSD) analyses. Low-cycle fatigue experiments under strain-controlled conditions were conducted in order to calibrate material property parameters for both an anisotropic crystal plasticity and an isotropic *J*_2_ model. Numerical finite element simulations were conducted using these synthetic microstructures and material properties based on experimental results, and cyclic stress-strain properties were calculated. Then, two-point correlations of synthetic microstructures were calculated to quantify the microstructures. The microstructure-property dataset was obtained by associating a two-point correlation and calculated cyclic stress-strain property. Machine learning, such as a linear regression model and neural network, was conducted using the dataset. Finally, cyclic stress-strain properties were predicted from the result of EBSD analysis using the obtained machine learning model and were compared with the results of the low-cycle fatigue experiments.

## 1. Introduction

It is important to consider fatigue problems when we design structural materials. It takes a considerable time and high cost to obtain enough information on the fatigue properties of new materials. Consequently, many researchers try to predict fatigue performance. As one way to predict material performance, machine learning, such as linear regression and neural network, has been attractive [1,2,3,4,5,6,7]. In these studies, prediction of performances, such as tensile strength and hardness, from chemical compositions and heat treatment conditions, was conducted. In many studies to predict material performance by machine learning, the performance is directly predicted from process conditions. However, in the case of complicated phenomena, such as fatigue, it is difficult to obtain a prediction model that can be applied to a wide range of materials. For example, a helpful prediction model for carbon steels is not always applicable to stainless steel [7].

Model-based machine learning [8] is one approach to predict complicated phenomenon. In model-based machine learning, one complicated phenomenon is divided into several elementary phenomena. This framework can be extended to more complex situations. In the case of material performance prediction, we can divide it into four stages: Process, structure, property, and performance [9]. Especially in the case of fatigue, there are various methods to connect process and structure such as phase filed method and Monte Carlo method. Moreover, fatigue life can be predicted from the cyclic stress-strain property by using the Tanaka-Mura model and finite element method (FEM) [10,11,12]. Therefore, we focused on the linkage between microstructure and cyclic stress–strain property in this study.

The microstructure-property data is not always sufficient for machine learning. Thus, a lot of finite element simulations are conducted instead of fatigue experiments to obtain the microstructure-property dataset. Also, it is necessary to quantify the microstructure for machine learning. Although there are various kinds of quantification methods, an appropriate method for fatigue prediction has not been established. We quantify microstructure by using one of the distribution functions: Two-point correlation [13]. The purpose of this study is to propose the method to predict cyclic stress–strain property from microstructure by combining finite element simulation, two-point correlation, and machine learning.

**Figure 1:**
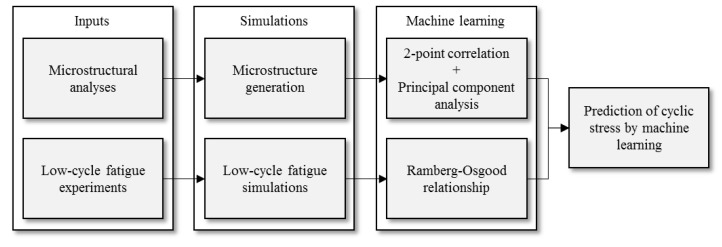
Framework to predict cyclic stress–strain property from microstructure by combining finite element simulation, two-point correlation, and machine learning.

Figure 1 shows the proposed framework. The framework can be divided into the following steps: (i) microstructure analysis with electron backscatter diffraction (EBSD) analysis, synthetic microstructure generation and two-point correlations, (ii) calibration of material parameters by strain-controlled low-cycle fatigue experiments, (iii) finite element simulations with the synthetic microstructure, and (iv) machine learning using two-point correlation and Ramberg–Osgood relationship. The rest of the paper is organized as follows. In Section 2, microstructure analysis and strain-controlled low-cycle fatigue experiments are presented. Finite element models are then created based on the results of the microstructure analysis, and parameters for simulation are identified from the results of fatigue experiments in Section 3. A set of finite element simulations is conducted, and stress–strain hysteresis loops are obtained. In Section 4, two-point correlations are calculated from finite element models, and the dimension of two-point correlation is reduced by principal component analysis (PCA). Then, machine learning is conducted with the obtained dataset in Section 5. The inputs are strain amplitude and principal components of two-point correlations, and the output is maximum stress (*σ*_max_) of each hysteresis loop. Finally, *σ*_max_ is predicted from the result of microstructure analysis by the obtained prediction model, and it is compared with the experimental result in Section 6. The validity of the proposed method for predicting cyclic stress–strain properties is discussed.

## 2. Experiments

### 2.1. Materials

Five types of low-carbon steel were used. Four were ferrite/pearlite dual-phase steels, and the other was pearlite single-phase steel. Table 1 shows the chemical composition of the steels. The chemical composition was measured on the rolled materials mainly by spark discharge optical emission spectrometry (SD-OES). The volume fraction of ferrite was calculated from the carbon contents (S25C: 71%, S35C: 60%, S45C: 40%, K1: 92%, Pearlite: 0%).

### 2.2. Microstructure Analysis

Each sample was cut, mechanically polished by emery paper and alumina slurry, and the cross-section was finally polished with an argon ion cross-section polisher (SM-090010, JEOL, Tokyo, Japan). EBSD analysis was performed for the four steels (S25C, S35C, S45C, and K1) to characterize the grain morphology. The observation area was approximately 330 × 540 ${\mu m}^{2}$ and the step size was 2 μm. To identify the ferrite and pearlite phases, image quality (IQ) was used. The IQ value of pearlite tends to be smaller than that of ferrite because pearlite is the lath structure of ferrite and cementite. In this research, we assumed that grain with large IQ value is ferrite, and grain with small IQ value is pearlite. Based on this assumption, the threshold of IQ value between ferrite and pearlite was determined so that the area fraction of the IQ map matched the volume fraction calculated in Section 2.

**Figure 2:**
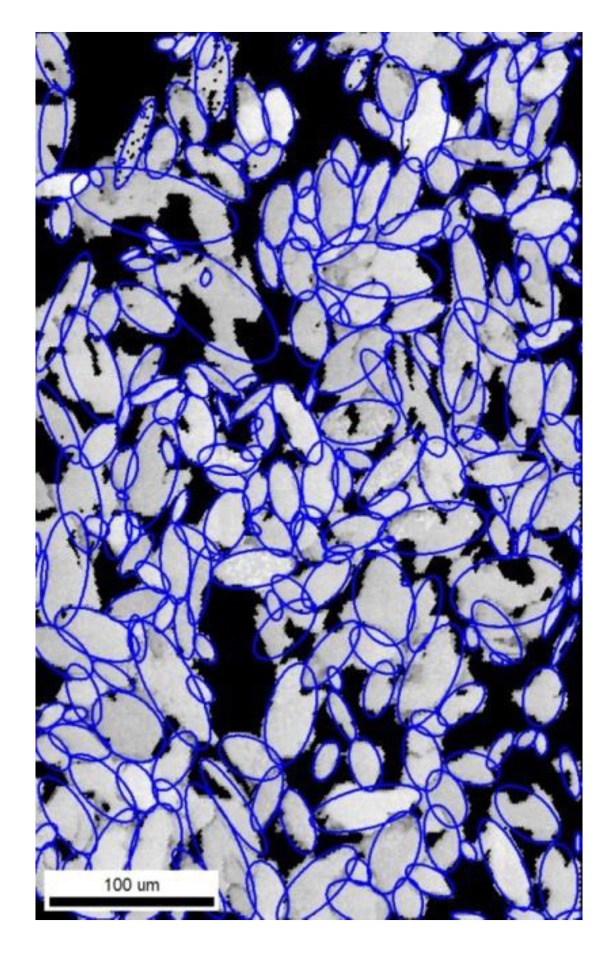
Image quality (IQ) map of S25C steel with ellipses fitted to ferrite grains.

After the phase identification, grains were fitted into ellipses that can be characterized by three parameters: the major axis a, the aspect ratio b/a (b is the minor axis) and the ellipse orientation angle θ with the horizontal direction. Phase identification and ellipse fitting were conducted using the software EDAX TSL OIM Analysis 7 (EDAX Inc., Mahwah, NJ, USA). Figure 2 shows the result of ellipse fitting for the ferrite grain of S25C steel. The black region represents the pearlite phase. The ellipse data were then fitted with probability distribution functions (PDF) by using MATLAB R2016a (Math Works Inc., Natick, MA, USA) distribution fitting toolbox. Since the major axis distribution exhibited a positive skewness, it was fitted with the log-normal distribution whose PDF is given by

(1) $$f\left(x\right)=\frac{1}{\sqrt{2\pi }\sigma_{\mathrm{SD}}x}\exp\left\{-\frac{{\left(\ln x-\mu \right)}^{2}}{2\sigma_{\mathrm{SD}}^{2}}\right\}$$

where μ and σ_SD_ are the location and scale parameters respectively. The distribution of the aspect ratio was almost symmetrical. The normal distribution was used as a fitting function. The PDF of the normal distribution is given by

(2) $$f\left(x\right)=\frac{1}{\sqrt{2\pi }\sigma_{\mathrm{SD}}}\exp\left\{-\frac{{\left(x-\mu \right)}^{2}}{2\sigma_{\mathrm{SD}}^{2}}\right\}$$

**Figure 3:**
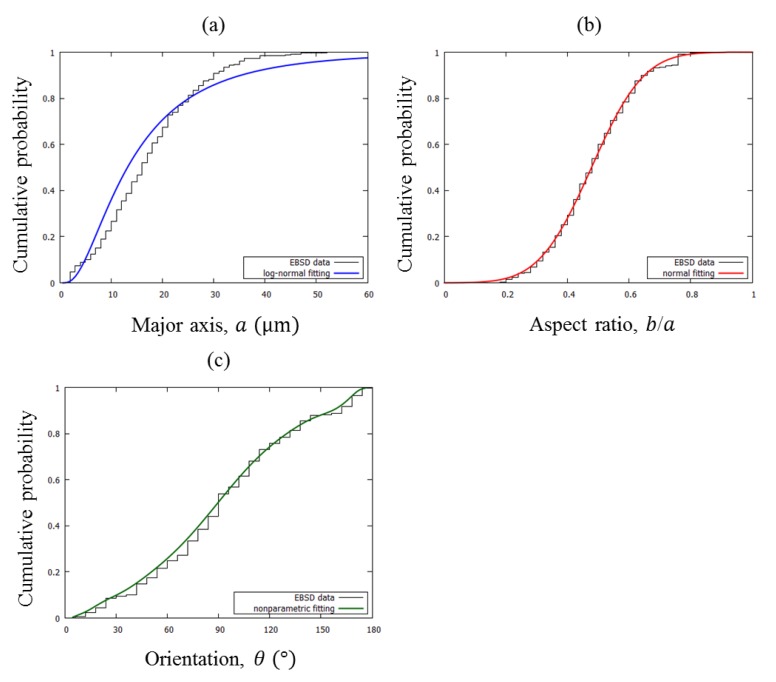
Cumulative distribution functions of (**a**) major axis, (**b**) aspect ratio, and (**c**) orientation for ferrite of S25C steel.

Since the ellipse orientation did not follow a particular distribution, it was fitted with a nonparametric distribution based on normal kernel smoothing. The fitted cumulative distribution functions and experimental data for ferrite grain of S25C are displayed in Figure 3. The microstructures of the other three steels (S35C, S45C, and K1) were also analyzed by the same procedure. Finally, four sets of probability distributions for grain morphology were obtained.

### 2.3. Low-Cycle Fatigue Experiments

**Figure 4:**
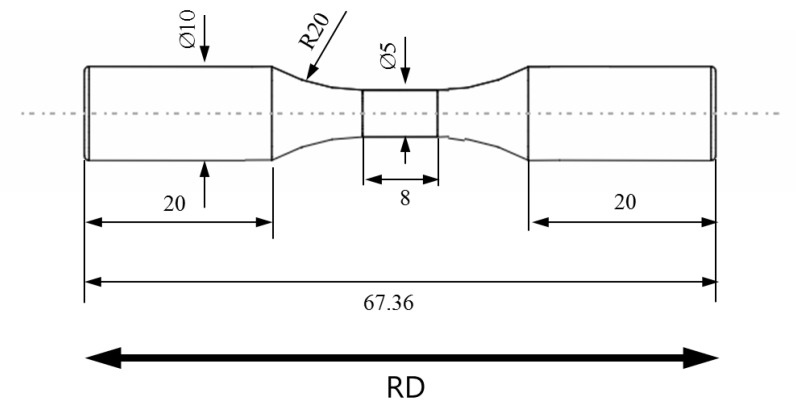
Geometry of smooth specimens used for low-cycle fatigue experiments (dimension in mm).

**Figure 5:**
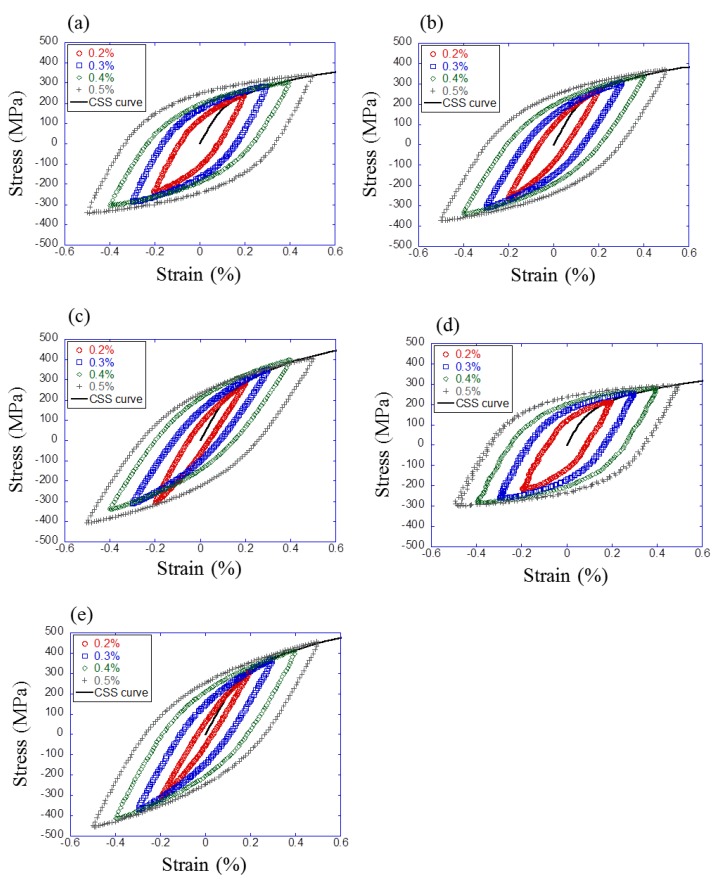
Experimental stress–strain hysteresis loop and cyclic stress–strain (CSS) curve for (**a**) S25C, (**b**) S35C, (**c**) S45C, (**d**) K1, and (**e**) Pearlite steels.

Strain-controlled low-cycle fatigue experiments were conducted to characterize the mechanical behavior of five materials under cyclic loading. Figure 4 shows the shape of specimens in the experiments. All experiments were conducted with a constant strain rate of $0.002{\;s}^{-1}$ and four different strain amplitudes of 0.2%, 0.3%, 0.4%, and 0.5% on a 50 kN tension-compression testing machine (Servopulser 50 kN, Shimadzu, Kyoto, Japan). The frequency was set to a different value for each strain amplitude to keep the constant strain rate. The strain was measured using an extensometer with a gauge length of 8 mm (3442-008M-005M-ST, Epsilon Technology Corp., Jackson, WY, USA). The stabilized stress–strain hysteresis loops were extracted at half of fatigue life. The results are shown in Figure 5. In each figure, four hysteresis loops with different strain amplitudes were plotted. Also, the cyclic stress–strain (CSS) curve was calculated by the Ramberg–Osgood relationship [14] using the maximum stress of each hysteresis loop. The curve is referred to as the “cyclic flow curve” in the literature. The Ramberg–Osgood relationship is defined as follow

(3) $$\varepsilon =\varepsilon_{e}+\varepsilon_{p}=\frac{\sigma }{E}+{\left(\frac{\sigma }{K}\right)}^{\frac{1}{n}}$$

where K and n are material constants, and E is Young’s modulus. In this study, Young’s modulus was 210 GPa. The obtained constants of Ramberg–Osgood relationship are shown in Table 2. These results were used to identify the material parameters for finite element simulations.

## 3. Finite Element Analysis of Low-Cycle Fatigue

### 3.1. Generation of Synthetic Microstructure

The generation of synthetic microstructures can be conducted based on various tessellation methods. In this study, microstructures were created by an anisotropic tessellation method [11] to reproduce the detailed grain morphology. In the method, ellipses were sampled until the total area occupied by the ellipse exceeds a given threshold:

(4) $$\pi \overset{n}{\underset{i=1}{\sum }}a_{i}b_{i}\geq \left(1+\lambda \right)A$$

where $a_{i}$ and $b_{i}$ respectively correspond to the major and minor axis of the ellipse i randomly extracted from the probability distribution functions, λ is an efficiency factor set to 20%, and A is the total area. In the reference to [11], single-phase microstructures were created. In order to generate dual-phase microstructures, Equation (4) was rewritten to Equation (5).

(5) $$\pi \overset{n}{\underset{i=1}{\sum }}a_{i}b_{i}\geq \left(1+\lambda \right)VA$$

where V is the volume fraction of each phase.

**Figure 6:**
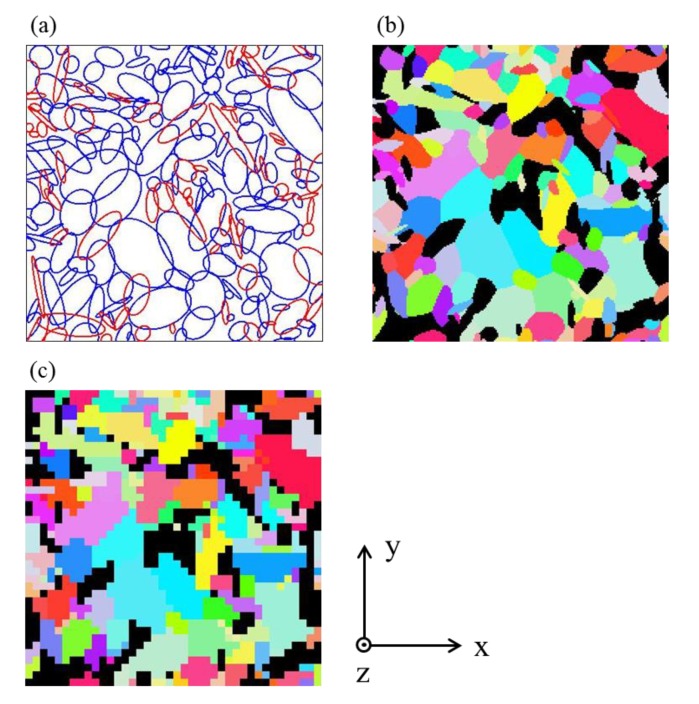
(**a**) Final configuration of the ellipse fitting process, (**b**) results of the anisotropic tessellation, and (**c**) finite element model.

After the ellipse sampling, the ellipses were positioned in the domain using a relaxed random sequential addition (RSA) algorithm [15]. The ellipses were allowed overlapping with the condition:

(6) $$\frac{{\left(x-x_{i}\right)}^{2}}{a^{2}}+\frac{{\left(y-y_{i}\right)}^{2}}{b^{2}}>{\left(1+\beta \right)}^{2}$$

where β is an overlap percentage proposed by St-Pierre et al. [16] and was set to 20%. The domain was set to 200 × 200 ${\mu m}^{2}$. Figure 6a shows the example of the final configuration of the ellipse filling process by the relaxed RSA algorithm. The blue and red ellipse show ferrite and pearlite grains, respectively.

Anisotropic tessellation was conducted for the obtained ellipses. The anisotropic metric function is as follow:

(7) $$d_{i}\left(x,x_{i}\right)=\left\|W_{i}\left(x-x_{i}\right)\right\|$$

where the anisotropic weight matrix $W_{i}$ is given by:

(8) $$W_{i}=\left(\begin{array}{cc} \frac{\cos\theta_{i}}{a_{i}} & \frac{\sin\theta_{i}}{a_{i}}\\ -\frac{\sin\theta_{i}}{b_{i}} & \frac{\cos\theta_{i}}{b_{i}} \end{array}\right)$$

where $a_{i},\;b_{i},\;\theta_{i}$ are the major axis, minor maxis, and orientation of the ellipse i, respectively? Figure 6b shows the result of anisotropic tessellation from Figure 6a. The black grains are pearlite, and the colored grains are ferrite. After the tessellation, a finite element model was created based on the synthetic microstructure. The mesh size was 5 × 5 ${\mu m}^{2}$ (totally 40 × 40 = 1600 elements per microstructure). Figure 6c shows the finite element model derived from Figure 6b.

### 3.2. Constitutive Models

Two types of constitutive models were used to reproduce some basic phenomena observed experimentally: Isotropic hardening, kinematic hardening and cyclic hardening/softening. One is macroscopic $J_{2}$ model and the other is a crystal plasticity (CP) model. The $J_{2}$ model is based on the second invariant of the deviatoric stress $J_{2}$. The yield function is defined by

(9) $$f=\sqrt{3J_{2}\left(s-\alpha^{dev}\right)}-\sigma_{y}=\sqrt{\frac{3}{2}{\left(\sigma -\alpha \right)}^{dev}:{\left(\sigma -\alpha \right)}^{dev}}-\sigma_{y}$$

where σ is the macroscopic stress tensor, α is the kinematic back stress tensor and $\sigma_{y}$ is the yield stress. Isotropic hardening is modeled as follow [17]

(10) $$\sigma_{y}=\sigma_{0}+Q_{\infty }\left(1-e^{-b{\bar{\varepsilon }}^{pl}}\right)$$

where $\sigma_{0}$ is the yield stress at zero strain, $Q_{\infty }$ is the maximum change in the yield stress, b is the rate of hardening and ${\bar{\varepsilon }}^{pl}$ is the equivalent plastic strain. On the other hand, kinematic back stress is defined as follow [18]

(11) $$\alpha =\overset{N}{\underset{k=1}{\sum }}\alpha_{k}$$

(12) $${\dot{\alpha }}_{k}=\frac{C_{k}}{\sigma_{y}}\left(\sigma -\alpha \right){\dot{\bar{\varepsilon }}}^{pl}-\gamma_{k}\alpha_{k}{\dot{\bar{\varepsilon }}}^{pl}$$

where N is the number of back stress $\alpha_{k}$, and $C_{k}$ and $\gamma_{k}$ are material parameters. This model is already implemented in the FEM software Abaqus. In this study, the $J_{2}$ model was used to analyze the plastic deformation of pearlite grains.

**Figure 7:**
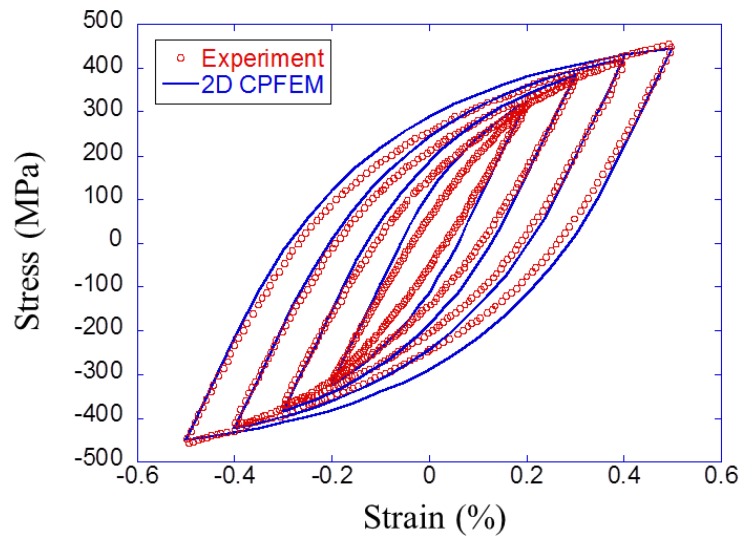
Experimental and calibrated stress–strain hysteresis loop for pearlite steel.

The 100% pearlite finite element models were created and used for parameter identification. The parameters of $J_{2}$ model were calibrated by using the optimization module of Abaqus software so that simulation results of hysteresis loops in five cycles reach experimental results for pearlite steel. Table 3 summarizes the parameters and Figure 7 shows the simulated hysteresis loops in five cycles compared with the experimental stable hysteresis loop for the pearlite steel. The Young’s modulus and Poisson’s ratio were set to E=210 GPa and ν=0.3, respectively.

The CP model considers plastic anisotropy for each grain. In this study, cubic elasticity following Hooke’s law and characterized by three coefficients, $C_{1111},\;C_{1122}$, and $C_{1212}$ was considered. The phenomenological constitutive law from the Damask user material subroutine for Abaqus [19] was used to analyze the plastic deformation in the ferrite grains. In the CP model, the crystal deformation gradient F is multiplicatively decomposed into elastic and plastic part,

(13) $$F=F_{e}F_{p}$$

The deformation velocity gradient L is defined as

(14) $$L=\dot{F}F^{-1}$$

By combining Equations (13) and (14), the velocity gradient can be expressed by

(15) $$L=\dot{F_{e}}F_{e}^{-1}+F_{e}\left(\dot{F_{p}}F_{p}^{-1}\right)F_{e}^{-1}=L_{e}+F_{e}\left(L_{p}\right)F_{e}^{-1}.$$

Assuming that plastic deformation occurs due to only dislocation slip from the $\left\{110\right\}\langle \bar{1}11\rangle$ slip system, the plastic velocity gradient $L_{p}$ is expressed as follow [20]

(16) $$L_{p}=\overset{N}{\underset{\alpha =1}{\sum }}{\dot{\gamma }}^{\alpha }\left(m^{\alpha }⊗n^{\alpha }\right)$$

where ${\dot{\gamma }}^{\alpha },\;m^{\alpha }$ and $n^{\alpha }$ are plastic share rate, slip direction vector, and normal to the slip plane of the slip system α, respectively. The N is the number of slip systems and was set to 12. Plastic shear rate is expressed by using resolved shear stress (RSS) and critical resolved shear stress (CRSS) [21,22,23,24]

(17) $${\dot{\gamma }}^{\alpha }={\dot{\gamma }}_{0}{\left|\frac{\tau^{\alpha }-\chi^{\alpha }}{\tau_{c}^{\alpha }}\right|}^{n}sgn\left(\tau^{\alpha }-\chi^{\alpha }\right)$$

where $\tau^{\alpha }=S:\left(m^{\alpha }⊗n^{\alpha }\right)$ and $\tau_{c}^{\alpha }$ are the RSS and CRSS on the slip system α, respectively. The back stress $\chi^{\alpha }$ is incorporated in order to consider kinematic hardening effects. Work hardening is expressed by increasing the CRSS on each slip system, as proposed by Bronkhorst et al. [25]:

(18) $$\tau_{c}^{\alpha }=\overset{N}{\underset{\beta =1}{\sum }}h^{\alpha \beta }\left|{\dot{\gamma }}^{\beta }\right|$$

with the hardening matrix $h^{\alpha \beta }$ defined by

(19) $$h^{\alpha \beta }=q^{\alpha \beta }h_{0}{\left(1-\frac{\tau_{c}^{\beta }}{\tau_{c_{s}}}\right)}^{a}$$

where α and β areslip systems, $q^{\alpha \beta }=1$ for coplanar slip systems while $q^{\alpha \beta }=1.4$ for non-coplanar systems. The $h_{0},\;a$ and $\tau_{c_{s}}$ are the hardening coefficient, the hardening exponent, and the saturated CRSS, respectively. Finally, kinematic hardening is modeled following Frederik–Armstrong law [18]

(20) $${\dot{\chi }}^{\alpha }=A{\dot{\gamma }}^{\alpha }-B\left|{\dot{\gamma }}^{\alpha }\right|\chi^{\alpha }.$$

### 3.3. Parameter Identification

**Figure 8:**
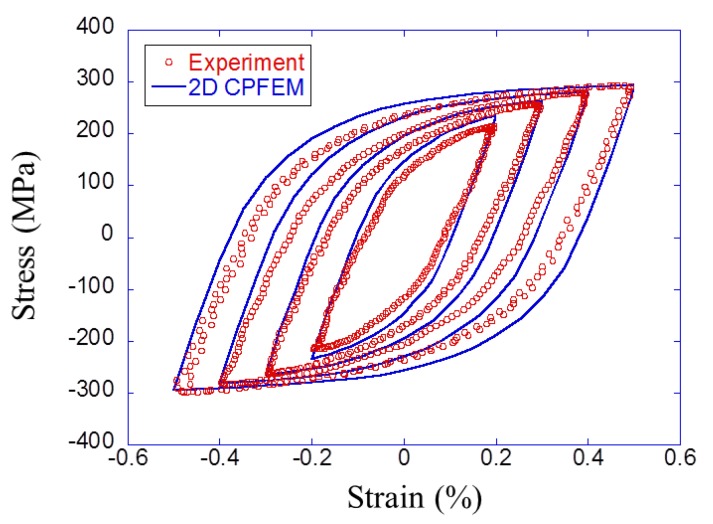
Experimental and calibrated stress–strain hysteresis loop for K1 steel.

In order to identify crystal plasticity parameters for ferrite grains, a finite element model of K1 steel was created based on the probability distributions of the K1 steel and volume fraction of 92%. The detailed identification method is written in the literature [11]. Since the K1 steel is a ferrite–pearlite steel, material constants of pearlite in Table 3 were used for calculating the plastic deformation in pearlite grains. Table 4 summarizes the crystal plasticity parameters, and Figure 8 shows the simulated hysteresis loops in five cycles compared with the experimental stable hysteresis loop for K1 steel. The elastic coefficients were taken from the literature [26]. These materials exhibit rate-dependent plasticity behavior. However, the predictions for different strain rates are beyond the scope of this paper. The numerical models were loaded at the same strain rate (0.002 s^−1^) as the experiments.

### 3.4. Low-Cycle Fatigue Simulations

Low-cycle fatigue simulations were conducted by FEM. The distribution of major axis and aspect ratio, the volume fraction of each phase, and the crystal orientation were necessary to create finite element models by the method explained above. The probability distributions for S25C, S35C, S45C, and K1 steels obtained in Section 2 were used to create models. The volume fraction of the ferrite phase was changed under ten conditions (10%, 20%, 100%). In the crystal orientation, three Euler angles $\left(\varphi_{1},\;\phi ,\varphi_{2}\right)$ were randomly assigned. Under the same conditions of the probability distributions, volume fraction, and orientation, different microstructures are created due to the randomness of ellipse sampling and positioning. To increase the number of data, five models were created for each condition. Totally 200 models (four grain morphologies × ten volume fractions × five models) were created.

Periodic boundary conditions were applied in the x and y directions, and analysis was carried out under plane strain condition. Strain-controlled fatigue simulations for y-direction were conducted with the same condition as experiments (strain rate of $0.002{\;s}^{-1}$, strain amplitudes: 0.2%, 0.3%, 0.4%, and 0.5%). The number of cycles was set to five.

**Figure 9:**
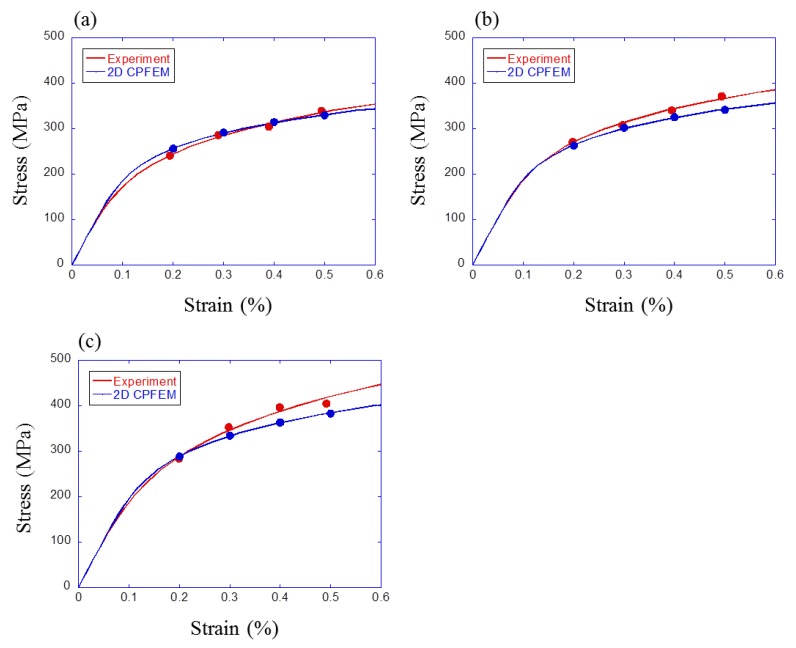
Experimental and simulated cyclic stress–strain curve for (**a**) S25C, (**b**) S35C, and (**c**) S45C.

In total, 800 crystal plasticity finite element method (CPFEM) analyses (200 models × four strain amplitudes) were conducted in this study. Among these results, results of models that reproduce the actual materials for both microstructure and volume fraction about S25C, S35C, and S45C steels were compared with experimental results, as shown in Figure 9. Each point is maximum stress in the hysteresis loop at half-life for the experiment and five cycles for simulation. Each curve is the Ramberg–Osgood cyclic stress–strain curve calculated with four maximum stresses. Since the model reproducing the volume fraction of S25C steel (ferrite volume fraction is 71%) was not created, Figure 9a shows the comparison result between experimental results for S25C steel and simulated results for the model with the ferrite volume fraction of 70%. The discrepancy between the experiments and simulations increased with the carbon content. The main reason is that the crystal plasticity parameters were calibrated with the polycrystalline model with lower carbon content. In order to realize a more accurate prediction, it is necessary to improve the microstructure reconstruction using a finer mesh and to examine a parameter calibration method in the future works.

## 4. Microstructure Quantification by Two-Point Correlations

**Figure 10:**
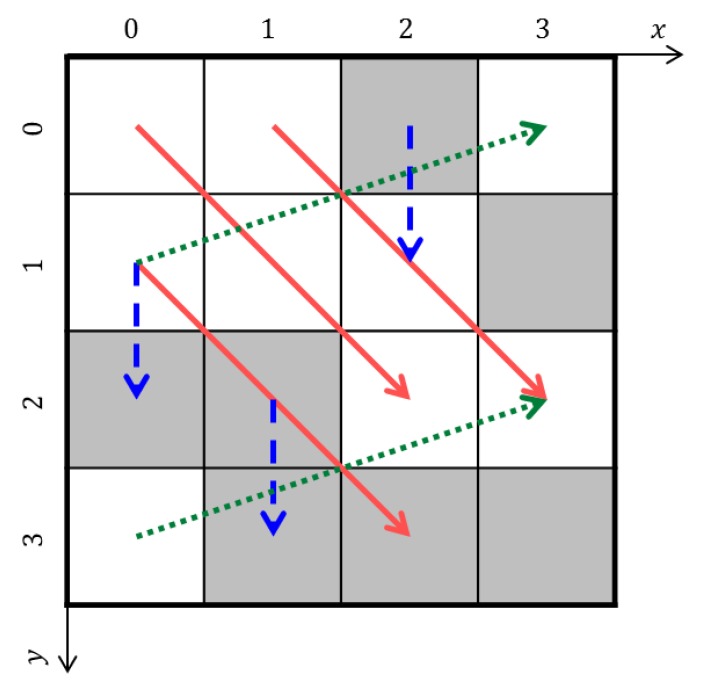
Illustration of two-point correlation.

Two-point correlation is one of the correlation functions. In recent years, Kalidindi and co-workers have presented the mathematical framework for quantification of microstructure based on a two-point correlation [27,28,29,30]. In this study, the two-point correlation of discretized microstructure was used to quantify microstructure. At first, the discretized microstructure was characterized with microstructure function $m_{s}^{n}$, where s is a spatial location and n is a local state. The microstructure function $m_{s}^{n}$ is the probability that the local state of location s is n. $m_{s}^{n}$ possess the following properties:

(21) $$\overset{N}{\underset{n=1}{\sum }}m_{s}^{n}=1,\;m_{s}^{n}\geq 0$$

where N is the number of possible local states. One-point correlation is defined by

(22) $$f^{n}=\frac{1}{S}\overset{S-1}{\underset{s=0}{\sum }}m_{s}^{n}$$

where S is the number of grid points. This one-point correlation corresponds to the volume fraction. In a similar manner, two-point correlation is defined by

(23) $$f_{t}^{nn^{\prime }}=\frac{1}{S}\overset{S-1}{\underset{s=0}{\sum }}m_{s}^{n}m_{s+t}^{n^{\prime }}$$

where t is the vector between two points. Figure 10 shows a simple example of a two-point correlation. Let n=1 for the white cell and n=0 for the gray cell. The microstructure functions are calculated like $m_{\left(1,0\right)}^{1}=1$, $m_{\left(3,1\right)}^{1}=0$ and $m_{\left(2,3\right)}^{0}=1$. One-point correlation (volume fraction) is also calculated as $f^{1}=9/16$ and $f^{2}=7/16$. The red arrows mean t=(2,2), blue arrows mean t=(0,1) and green arrows mean t=(3,−1). In general, a periodic boundary condition was applied to microstructure to calculate the two-point correlation. The two-point correlations have the following properties:

(24) $$f_{t}^{nn^{\prime }}=f_{t\pm S}^{nn^{\prime }}$$

(25) $$f_{t}^{nn^{\prime }}=f_{-t}^{n^{\prime }n}.$$

The vector t can take a value between $-\left(x-1\right)\leq t_{x}\leq x-1,\;-\left(y-1\right)\leq t_{y}\leq y-1$ for x×y domain. By using Equation (24), two-point correlation is defined in the following range:

(26) $$\begin{array}{cc} -\frac{S-1}{2}\leq t\leq \frac{S-1}{2} & \left(S\;\mathrm{is}\;\mathrm{odd}\;\mathrm{number}\right),\\ -\frac{S}{2}\leq t\leq \frac{S}{2} & \left(S\;\mathrm{is}\;\mathrm{even}\;\mathrm{number}\right). \end{array}$$

For dual-phase microstructure (n=0, 1), four two-point correlations, $f_{t}^{00},\;f_{t}^{11},\;f_{t}^{01},\;f_{t}^{10}$ can be calculated. However, only N−1 two-point correlations are independently defined for N phase microstructure by various interrelationships [27]. Therefore, only one two-point correlation $f_{t}^{FF}$. (ferrite–ferrite) was used to quantify ferrite-pearlite microstructures.

The two-point correlations were calculated using a fast Fourier transform (FFT) [27,29,30]. At first, a discrete Fourier transform (DFT) is conducted on Equation (23)

(27) $$F_{k}^{nn^{\prime }}=ℱ\left(f_{t}^{nn^{\prime }}\right)=\frac{1}{S}\overset{S-1}{\underset{t=0}{\sum }}\overset{S-1}{\underset{s=0}{\sum }}m_{s}^{n}m_{s+t}^{n^{\prime }}e^{-i\frac{2\pi kt}{S}}=\frac{1}{S}\overset{S-1}{\underset{s=0}{\sum }}m_{s}^{n}\overset{S-1}{\underset{t=0}{\sum }}m_{s+t}^{n^{\prime }}e^{-i\frac{2\pi kt}{S}}$$

where ℱ means DFT. Now let s+t=z, then

(28) $$F_{k}^{nn^{\prime }}=\frac{1}{S}\overset{S-1}{\underset{s=0}{\sum }}m_{s}^{n}\overset{S-1+s}{\underset{z=s}{\sum }}m_{z}^{n^{\prime }}e^{-i\frac{2\pi k\left(z-s\right)}{S}}=\frac{1}{S}\overset{S-1}{\underset{s=0}{\sum }}m_{s}^{n}e^{-i\frac{2\pi k\left(-s\right)}{S}}\overset{S-1+s}{\underset{z=s}{\sum }}m_{z}^{n^{\prime }}e^{-i\frac{2\pi kz}{S}}.$$

Finally, by periodic boundary condition, $m_{z}^{n^{\prime }}=m_{z+S}^{n^{\prime }}$, we can get the following equation

(29) $$F_{k}^{nn^{\prime }}=\frac{1}{S}\overset{S-1}{\underset{s=0}{\sum }}m_{s}^{n}e^{-i\frac{2\pi k\left(-s\right)}{S}}\overset{S-1}{\underset{z=0}{\sum }}m_{z}^{n^{\prime }}e^{-i\frac{2\pi kz}{S}}=\frac{1}{S}{\left(M_{k}^{n}\right)}^{*}M_{k}^{n^{\prime }}$$

where $M_{k}^{n}=ℱ\left(m_{s}^{n}\right)$ and * is the complex conjugate. Two-point correlation is easily obtained from Equation (29) by inverse DFT of $F_{k}^{nn^{\prime }}$.

In this study, two-point correlations $f_{t}^{FF}$ for 200 finite element models were calculated with the FFT method. Since the whole data of the two-point correlation was large for machine learning, principal component analysis (PCA) was conducted to reduce the dimension of two-point correlations. The 15 principal component scores of the two-point correlation were used for machine learning. The two-point correlation calculations with FFT and PCA were conducted with MATLAB R2016a.

## 5. Prediction of Cyclic Stress–Strain Property by Machine Learning

### 5.1. Linear Regression Model

Linear regression is one way of regression analysis in which the relationship between one or more parameters is modeled by a linear regression equation. In general, the linear regression model can be expressed as

(30) $$y=\overset{n}{\underset{i}{\sum }}a_{i}x_{i}+b$$

where y and $x_{i}$ are input and output value, n is the number of inputs, $a_{i}$ is the coefficient and b is the constant. We obtained the coefficients and constant using MATLAB R2016a ‘regress’ function. The algorithm of this function is written in the literature [31].

Generally in linear regression models, the more complex the model becomes, the more accurate the prediction becomes. However, if the model is too complex, the model becomes over-fitted, and the prediction for unknown data does not go well [32]. In this study, parameter selection from all $2^{15}-1$ combination of 15 principal components was conducted so that the model became the most accurate. Moreover, cross validation was conducted to reduce the error depending on how to divide data into training and test set. In this study, 700 training data was divided into 70 groups of 10 data for the cross validation. We used RMSE (root mean squared error, Equation (31)) as the criteria for the model selection.

(31) $$\mathrm{RMSE}=\sqrt{\frac{1}{N}\overset{N}{\underset{i=1}{\sum }}{\left(y_{i}-\hat{y_{i}}\right)}^{2}}$$

where N is the number of data, $y_{i}$ is the experimental (or simulated by FEM) value and $\hat{y_{i}}$ is the predicted value. The cross validation was conducted for all $2^{15}-1$ combinations of principal components and the combination with the smallest RMSE was used as the most accurate principal components. Finally, by using these components, coefficients and constants were calculated with all 700 training data and the obtained model was tested for 100 test data. Details of the procedure can be found in reference [7].

### 5.2. Neural Network Model

An artificial neural network is one way of machine learning which is inspired by biological neural networks. It can approximate almost any function by adjusting the weights and biases of each layer and unit. The general feedforward neural network consists of three layers: Input, hidden, and output layer. In each unit in hidden layers, calculation, as shown in Equation (32), is conducted and the result $h_{j}$ is sent to the next layer.

(32) $$h_{j}=\varphi \left(\overset{I}{\underset{i=1}{\sum }}w_{ij}x_{i}+b_{j}\right)$$

where $x_{i}$ is the input value from ith unit in the former layer, I is the number of units in the former layer, $w_{ij}$ and $b_{j}$ are weight and bias, and φ is a differentiable non-linear function called “activation function.” In the output unit, identity function φ(x)=x is generally used as the activation function. Thus, the output is calculated as

(33) $$o=\overset{J}{\underset{j=1}{\sum }}w_{j}h_{j}+b_{o}$$

where J is the number of the last hidden units.

Backpropagation [33] is widely used to set weights and biases suitably in neural network training. Weights and biases are adjusted to minimize the sum of squared of error between dataset and predicted value as

(34) $$E=\overset{N}{\underset{k=1}{\sum }}{\left(y_{k}-o_{k}\right)}^{2}$$

where $y_{k}$ and $o_{k}$ are dataset and predicted value, respectively. Various calculation procedures for the error reduction of backpropagation have been proposed, such as the steepest descent method and the Gauss–Newton method. Levenberg–Marquardt method [34,35] is the combination of the steepest descent method and the Gauss–Newton method and can finish training fast and accurately.

As mentioned above, neural networks can approximate almost any function. In other words, it is important to be careful with over-fitting. The Bayesian framework is widely studied to control this problem [2,32,36]. In a neural network with Bayesian framework, weights and biases are adjusted to minimize the following function instead of E in Equation (34):

(35) $$M\left(w\right)=\beta E_{D}+\alpha E_{w}$$

(36) $$E_{D}\left(w\right)=\frac{1}{2}\sum_{n=1}^{N}{\left(y_{n}-o_{n}\right)}^{2},\;E_{w}\left(w\right)=\frac{1}{2}w^{⊤}w.$$

where $E_{D}$ is the same as Equation (34). $E_{w}$ is the sum of squared of weights and it works to reduce the value of weights. Two parameters α and β are at first constant parameters corresponding to the variance of weights and data in the beginning, respectively. The α and β are updated during the training process. The parameter adjusting process is a substitute for validation data. Thus only training and test data are necessary for a neural network with Bayesian framework. In MATLAB R2016a Neural Network Toolbox, various algorithms for neural network training are prepared. In this study, we used the Levenberg–Marquardt method with a Bayesian framework from these prepared algorithms.

Various activation functions have been used in neural networks. Sigmoid function (Equation (37)) was used commonly at the beginning of neural network study because it is simple and easy to differentiate.

(37) $$\varphi \left(x\right)=\frac{1}{1+e^{-x}}.$$

After that, Glorot and Bengio [37] suggested that hyperbolic tangent (Equation (38)) is better and later, Glorot et al. [38] presented that Rectified Linear Unit (ReLU, Equation (39)) is better than hyperbolic tangent. LeCun et al. [39] reported in 2015 that ReLU is the best activation function. However, ReLU is a very simple function and expressing ability by itself is poor and is not necessarily the best activation function for the network with few hidden layers.

(38) $$\varphi \left(x\right)=\tanh\left(x\right)=\frac{e^{x}-e^{-x}}{e^{x}+e^{-x}}$$

(39) φ(x)=max(0,x).

In this study, hyperbolic tangent and ReLU were compared.

In general, input and output values are normalized before training to eliminate the deviation of data. In this study, both the input and output variables except strain amplitude were normalized so that the average becomes zero and the variance becomes one (standardization) as follows:

(40) $$x\prime_{i}=\frac{x_{i}-\bar{x}}{s}$$

where $x_{i}$ is the input/output value of ith data, $x\prime_{i}$ is the normalized value and $\bar{x}$ and s are the average and standard deviation.

The training of the neural network with multi hidden layers is called “deep learning.” The improvement of computer performance and a vast amount of data obtained by the development of the Internet boost the research of deep neural networks. It is considered that prediction accuracy is improved with deep learning, but the general deep learning is conducted with more than thousands of data. Only 800 data is available in this study, and it is not appropriate to use deep learning in this case. Thus, the number of hidden layers was set to only 1 or 2 in this study. Moreover, the number of units in hidden layers may affect prediction accuracy. In this study, three conditions of hidden layers were compared.

- One hidden layer with five units
- One hidden layer with ten units
- Two hidden layers with ten and five units

Together with two activation functions, six neural networks were constructed and trained/tested with the same data. The network with the minimum RMSE value for test data was used as the best neural network model.

### 5.3. Microstructure-Property Dataset

In Section 4, 800 CPFEM analyses (200 models × 4 strain amplitudes) were conducted. The principal components of two-point correlations of 200 finite element models were calculated in Section 5. The 15 principal component scores and strain amplitude were set to input values. The calculated maximum stress $\sigma_{max}$ in five cycles was set to output value. We have 800 microstructure-property data in total. The data were sorted randomly and divided into two groups: 700 for training and 100 for testing. Although the dataset is recommended to divide three groups of training, validation, and testing [32], we used the machine learning algorithm that does not require a validation dataset.

### 5.4. Results of Machine Learning

**Figure 11:**
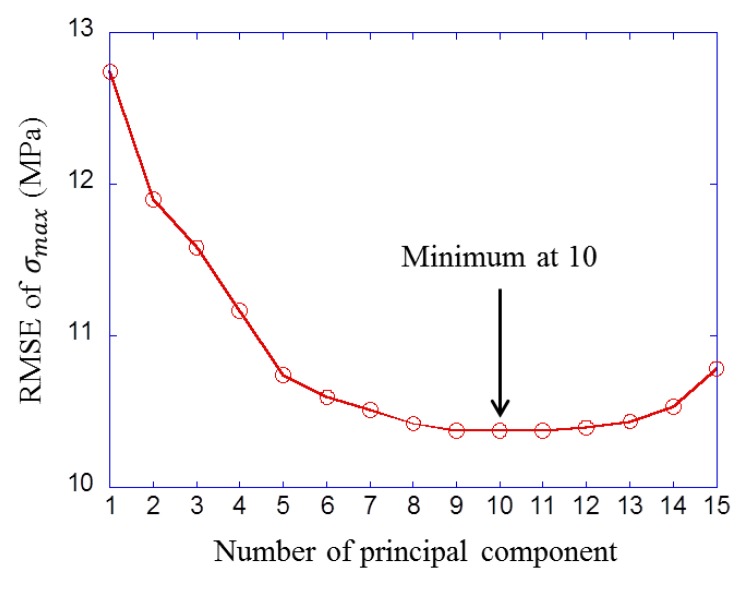
Result of parameter selection in linear regression model.

**Figure 12:**
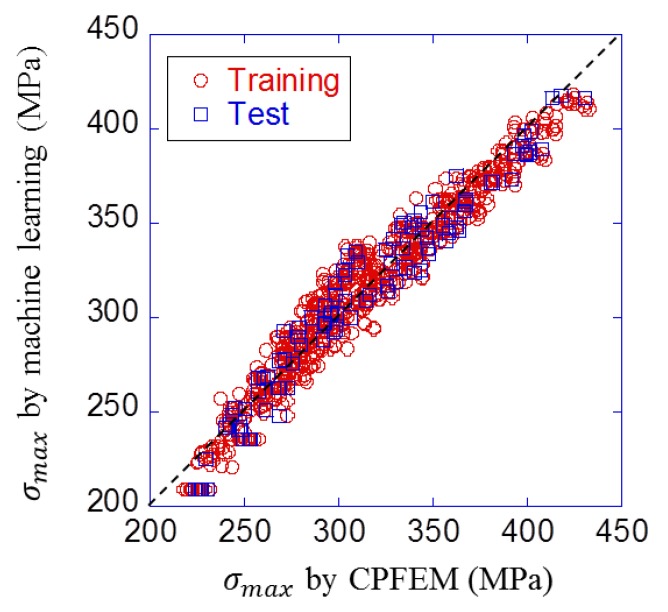
Prediction result of linear regression model with the best combination of principal components.

Figure 11 shows the result of parameter selection with the cross validation in the linear regression model. The horizontal axis is the number of principal components, and the vertical axis is the minimum RMSE value of the model of each number of parameters. It can be seen that RMSE reached a minimum when the model had ten principal components (1, 2, 6, 7, 8, 9, 11, 13, 14, and 15th principal component). Figure 12 shows the prediction result with these ten principal components. The horizontal axis is the maximum stress $\sigma_{max}$ calculated by CPFEM (dataset value) and the vertical axis is $\sigma_{max}$ predicted by regression formula. This figure means that the prediction accuracy is higher as the plot is closer to the straight line. The RMSE was 11.6 MPa for training data and 12.1 MPa for test data.

**Figure 13:**
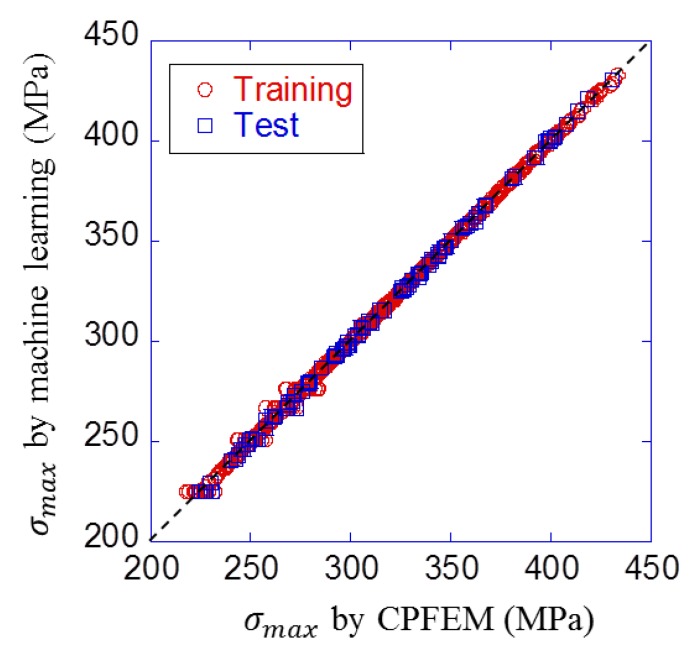
Prediction result of neural network model under the best condition.

Table 5 shows the comparison result of six neural network conditions. RMSE for test data became minimum value when the activation function was hyperbolic tangent, and the number of hidden layers was two with ten and five units. The prediction result with this condition is shown in Figure 13. It can be visually confirmed that plots are closer to the straight line than those of the linear regression model in Figure 12. Also, RMSE was 1.38 MPa for training data and 1.37 MPa for test data. It is clear both visually and quantitatively that the neural network model was more accurate than the linear regression model.

**Figure 14:**
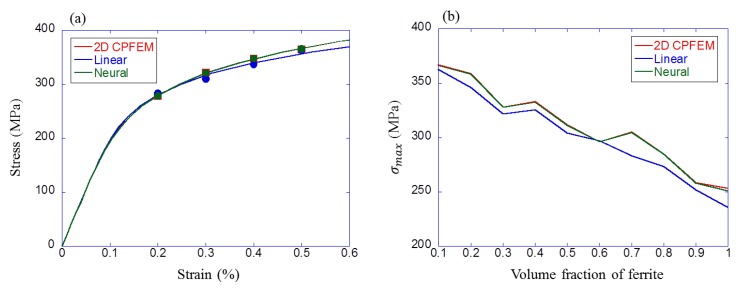
Comparison results between simulation and machine learning. (**a**) Only strain amplitude was changed with fixed grain morphology (K1) and ferrite volume fraction (50%), (**b**) only ferrite volume fraction was changed with fixed grain morphology (S25C) and strain amplitude (0.3%).

Moreover, these obtained models were compared with CPFEM results. Two comparisons were conducted by changing only one parameter. First, only strain amplitude was changed with fixed grain morphology (K1) and ferrite volume fraction (50%). The result is shown in Figure 14a. Cyclic stress–strain curves as Ramberg–Osgood relationships are calculated and also plotted. Second, only the ferrite volume fraction was changed with fixed grain morphology (S25C) and strain amplitude (0.3%). The result is shown in Figure 14b. As shown in these figures, machine learning, especially the neural network model, could predict the fatigue property accurately.

## 6. Discussion

### 6.1. Comparison of Simulation and Experimental Results

Experimental and simulated cyclic stress–strain curves were shown in Figure 9. As shown in the figure, the simulated results for S25C matched experimental results, but results for S35C and S45C did not match well. Since the mesh size was 5 μm in these simulations, the lower limit of grain size was 5 μm. However, the actual specimen, which was used for parameter identification, contains smaller grains than 5 μm. Thus, there is the possibility that the grain size in 2D modeling was overestimated. Since the parameters of the crystal plasticity model in ferrite grains were fitted to the K1 steel with the ferrite volume fraction of 92%, the prediction accuracy of the cyclic stress–strain curve decreased with increasing the divergence of volume fraction from K1. It seems that this was the reason that the simulated results for S25C matched experimental result but results for S35C and S45C did not.

There are two approaches to solve this problem. One is to use finer mesh, and the other is to modify the material parameters. The former method increases the calculation time and is not suitable for preparing a large dataset. The latter approach can provide a better fitting for S35C and S45C steels by adjusting the material property parameters. However, this approach may provide poor agreement with the experimental data of K1 and S25C steels. Therefore, identified parameters in this study were reasonable in case of analyzing various volume fractions with one material property.

### 6.2. Comparison of Machine Learning and Experimental Results

Finally, $\sigma_{max}$ was predicted from the result of microstructure analysis by the obtained prediction model, and it was compared with the experimental result. The square area of 200 × 200 ${\mu m}^{2}$ was trimmed from the result of EBSD analysis for S25C, S35C and S35C steels (e.g., Figure 2). These trimmed images were meshed by 5 μm and binarized to ferrite and pearlite. Then, two-point correlation $f_{t}^{FF}$ was calculated and principal component scores were calculated using principal component coefficient obtained from 200 finite element models. These 15 principal component scores and strain amplitude (0.2%, 0.3%, 0.4%, 0.5%) were substituted for obtained linear regression/neural network model and $\sigma_{max}$ was predicted. Finally, the cyclic stress–strain curve as the Ramberg–Osgood relationship was calculated and compared with experimental results.

**Figure 15:**
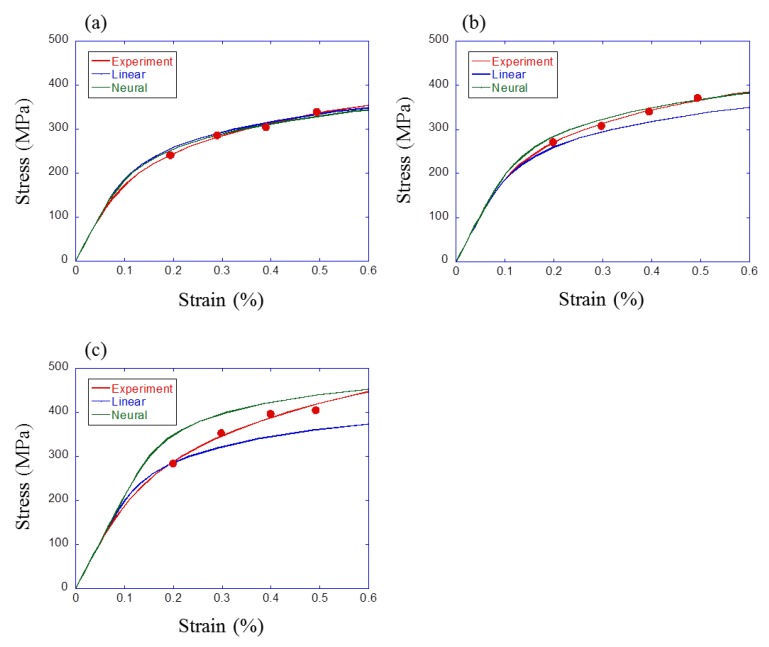
Comparison results between machine learning and experiment for (**a**) S25C, (**b**) S35C, and (**c**) S45C.

The results are shown in Figure 15. The red points are experimental $\sigma_{max}$ for each hysteresis loop and three curves are cyclic stress–strain curves. In the result for S25C (Figure 15a), there is no large difference between two prediction results and these results showed good agreement with the experimental result. On the other hand, in the result for S35C and S45C (Figure 15b,c), there is a difference between the prediction results of linear regression and the neural network model, and the experimental result lay between two prediction results. For S25C steel, there is a good agreement of experimental, simulated and predicted results. Therefore, the prediction method proposed in this study is effective for fatigue property prediction. As shown so far, cyclic stress–strain property could be predicted from microstructure. Recently, the Tanaka–Mura model has been modified and shown to be useful for the prediction of crack initiation in various metallic materials [40,41]. By combining the Tanaka–Mura model and CPFEM, as explained in our previous works [11,12], this prediction can be linked to fatigue life prediction.

## 7. Conclusions

In this study, the finite element method, two-point correlation and machine learning were combined to propose the new method to predict cyclic stress–strain property from the microstructure of ferrite-pearlite steel. Based on the experiment, simulation and prediction results and discussion presented in the preceding sections, the following conclusions were obtained.

- The result of the finite element analysis showed good agreement with the experimental results. The results confirmed that the material parameters identified in this study were appropriate for fatigue analysis.
- Cyclic stress–strain property of ferrite-pearlite steel could be predicted with high accuracy by combining two-point correlation and machine learning. Also, the prediction error of the neural network model was smaller than that of the linear regression model.
- Cyclic stress–strain property predicted from the result of microstructure analysis by the model obtained by machine learning showed a good agreement with the experimental results. Thus, the prediction method proposed in this study was shown to be effective for fatigue property prediction.

## Figures and Tables

**Table 1:** Chemical composition of steels (in mass%).

Steel	C	Si	Mn	P	S	Cu	Ni	Cr	Al
S25C	0.24	0.18	0.44	0.014	0.003	0.01	0.01	0.01	0.028
S35C	0.32	0.17	0.63	0.014	0.004	0.13	0.07	0.13	0.019
S45C	0.47	0.16	0.60	0.017	0.004	0.18	0.12	0.12	0.018
K1	0.07	<0.01	1.52	0.006	0.003	-	-	-	0.028
Pearlite steel	0.80	0.006	0.039	0.002	0.001	-	-	-	-

**Table 2:** Material constants of Ramberg–Osgood relationship.

Steel	*K*/MPa	*n*
S25C	1233	0.228
S35C	1170	0.202
S45C	1719	0.242
K1	1124	0.234
Pearlite steel	1626	0.219

**Table 3:** Finite element analysis parameters of pearlite.

Elasticity		Isotropic Hardening		
E (GPa)	ν	$\sigma_{0}\;\left(\mathrm{MPa}\right)$	$Q_{\infty }\;\left(\mathrm{MPa}\right)$	b
210	0.3	160	−40	7
Kinematic hardening (N=5), $C_{k}\;\left(\mathrm{GPa}\right)$				
$C_{1}$	$C_{2}$	$C_{3}$	$C_{4}$	$C_{5}$
37,671	34,881	59,554	62,858	351,000
$\gamma_{1}$	$\gamma_{2}$	$\gamma_{3}$	$\gamma_{4}$	$\gamma_{5}$
255.39	645.45	2014.6	1972.6	12,615

**Table 4:** Finite element analysis parameters of ferrite. Elastic coefficients are taken from the literature [26].

Cubic Elasticity					Kinematic Law	
$C_{1111}\;\left(\mathrm{GPa}\right)$	$C_{1122}\;\left(\mathrm{GPa}\right)$	$C_{1212}\;\left(\mathrm{GPa}\right)$			$\dot{\gamma_{0}}$	n
233.3	135.5	118.0			0.001	4
Work hardening					Kinematic hardening	
$\tau_{c_{0}}\;\left(\mathrm{MPa}\right)$	$\tau_{c_{s}}\;\left(\mathrm{MPa}\right)$	$h_{0}\;\left(\mathrm{MPa}\right)$	a	$q^{\alpha \beta }$	A (MPa)	B
50	110	150	2.25	1/1.4	26,000	1000

**Table 5:** RMSE (MPa) for test data in six neural network conditions.

		Activation Function	
		tanh	ReLU
**Hidden layer condition**	(i)	4.24	6.23
	(ii)	2.09	3.96
	(iii)	1.37	3.63
